# Geospatial Epidemiology in Low‐Resource Settings: Advances, Challenges, and Future Directions

**DOI:** 10.1155/ghe3/1751686

**Published:** 2026-11-09

**Authors:** Yusuf Hared Abdi, Sharmake Gaiye Bashir, Yakub Burhan Abdullahi, Mohamed Sharif Abdi, Naima Ibrahim Ahmed

**Affiliations:** ^1^ Department of Public Health Faculty of Medicine and Health Sciences Hormuud University Mogadishu Somalia trjums.ac.ir

**Keywords:** disease surveillance, geospatial epidemiology, health equity, low-resource settings

## Abstract

Geospatial epidemiology has emerged as a critical tool to strengthen disease surveillance, resource allocation, and health equity in low‐resource settings. This narrative review synthesizes evidence on the recent applications, methodological advances, and implementation challenges of geospatial approaches across infectious diseases, maternal and child health, noncommunicable diseases, climate‐sensitive conditions, and humanitarian contexts. Key sources, including GIS, remote sensing, population grids, Demographic and Health Surveys, and routine health information systems, have enabled increasingly refined spatial analyses, from malaria and cholera hotspot detection to accessibility modeling for maternal care and climate–health early warning systems. However, persistent limitations remain, including incomplete surveillance data, inaccurate or displaced geocoding, unstable population denominators, and constrained technical and institutional capacity, which restrict the consistent translation of spatial insights into policy actions. Ethical concerns related to data privacy and governance further complicate the use of high‐resolution mapping for vulnerable populations. We identify emerging opportunities to address these gaps, including the integration of artificial intelligence, strengthening of national spatial data infrastructure, community‐based geospatial data collection, and linkage with civil registration and identification systems under robust governance frameworks. Institutionalizing geospatial capacity within health systems is essential for transforming spatial analytics from episodic research exercises to a sustainable foundation for precision public health and equitable service delivery in low‐resource settings.

## 1. Introduction

Geospatial epidemiology examines geographic variation in disease burden and its environmental, demographic, behavioral, and socioeconomic determinants. It combines geographic information systems (GIS), remote sensing (RS), geocoded health data, and spatial statistical models to describe disease patterns, detect clusters, estimate risk, and guide interventions. Its relevance is especially clear in low‐ and middle‐income countries, where health risks and access to services vary markedly within countries [1–3].

In low‐resource settings (LRS), geospatial methods address practical weaknesses in surveillance and service delivery. Incomplete reporting, delayed notification, weak laboratory networks, and geographically aggregated data can conceal outbreaks and underserved communities. Climate shocks and population displacement further change transmission patterns and population distributions, creating a need for spatially adaptive surveillance [4–6]. Open geospatial resources have expanded the evidence available to health programs. Important sources include geocoded Demographic and Health Surveys and gridded population products such as WorldPop, satellite‐derived climate data, and spatially referenced routine health information systems. However, wider data availability has not resolved weaknesses in geocoding, analytical capacity, governance, or routine data use.

Three evidence gaps motivate this review. First, applications across different health domains are often discussed separately, limiting cross‐sectoral learning. Second, positional error, temporal mismatch, missing facility coordinates, and uncertain population denominators can weaken spatial inference. Third, limited institutional capacity and fragmented governance prevent useful analyses from becoming routine health‐system functions [7].

This review has three aims. First, it synthesizes major geospatial applications across infectious diseases, climate‐sensitive outcomes, maternal and child health (MCH), noncommunicable diseases (NCDs), and population mobility. Second, it assesses the principal data and methodological limitations. Third, it identifies priorities for sustainable, ethical, and country‐owned geospatial health intelligence.

## 2. Methods

This narrative review synthesized peer‐reviewed evidence on the applications, limitations, and implementation priorities of geospatial epidemiology in LRS. PubMed/MEDLINE, Scopus, Web of Science, and Google Scholar were searched in August 2026 for English‐language publications from January 2010 through August 2026. Search terms included “geospatial epidemiology,” “spatial analysis,” and “GIS.” These were combined with “low‐resource settings,” “low‐ and middle‐income countries,” “sub‐Saharan Africa,” or “South Asia.” We included original studies, reviews, guidelines, and consensus statements that applied or discussed geospatial methods for health outcomes in LRS. We excluded studies confined to high‐income settings without clear methodological or policy relevance. Sources were selected purposively to represent the disease domains, data sources, methods, and implementation challenges covered by the review. Evidence was organized thematically and synthesized narratively. No protocol registration, duplicate screening, or formal risk‐of‐bias assessment was undertaken. Accordingly, this review is interpretive rather than systematic or scoping. Table 1 summarizes the representative studies discussed in the applications section.

**TABLE 1:** Summarizes representative applications discussed in this section.

Application	Setting	Objective	Data source	Method	Key finding
Malaria mapping	Sub‐Saharan Africa	Estimate prevalence/incidence	DHS, MAP, environmental covariates	Bayesian geostatistics, random forest	High‐resolution risk surfaces guide net/spray targeting
TB hotspot mapping	Tanzania, high‐burden countries	Identify transmission clusters	Case notification, eLMIS data	Spatial autocorrelation	Clusters linked to underdiagnosis areas
Cholera clustering	DRC, conflict/postdisaster settings	Rapid outbreak detection	Case surveillance, satellite imagery	Spatial cluster analysis	Faster outbreak response in emergencies
Dengue risk mapping	Pakistan, Bangladesh, Thailand, Sri Lanka	Predict transmission risk	Climate data, case reports	ML (random forest, SVM), geographic profiling	Identified breeding‐site hotspots and hyperendemic zones
Polio/measles surveillance	Africa (AFRO)	Close immunization gaps	GIS Center vaccination data	Hotspot analysis	Identified under‐vaccinated pockets
Flood‐related disease	South Asia	Link floods to disease outbreaks	WorldPop, travel‐time data	Spatial overlay	Prioritized flood‐exposed, low‐access areas
Drought/malnutrition	Sahel, Horn of Africa	Map stunting/malnutrition risk	Household surveys	Spatial regression	Linked stunting to drought exposure
Heatwave mortality	Ethiopia	Identify heat‐vulnerable areas	Climate + health records	GWR	Found urban cardiovascular risk hotspots
Air pollution exposure	African/Asian cities	Estimate PM2.5 exposure	Satellite (MODIS, SEDAC)	Remote sensing modeling	Revealed pollution hotspots lacking ground monitors
Maternal/child health inequality	16 SSA countries	Measure service coverage gaps	DHS	Concentration index, Bayesian regression	Found wealth‐based inequality in maternal care
Health facility accessibility	Mozambique	Optimize facility placement	Facility location data	Nearest‐neighbor, location‐allocation	Identified underserved catchment areas
Hypertension/diabetes mapping	South Africa, Ethiopia, DRC	Map NCD burden	Population surveys	GWR	Found urban hypertension hotspots
Road traffic mortality	Bangladesh	Map accident mortality	Government records	District‐level geospatial analysis	Identified high‐mortality districts
Displacement surveillance	Uganda, Bangladesh, DRC	Track displaced populations	Satellite imagery, camp records	Satellite enumeration	Enabled real‐time population estimates

## 3. Applications of Geospatial Epidemiology in LRS

### 3.1. Infectious Disease Mapping: From Descriptive Cartography to Predictive Analytics

#### 3.1.1. Malaria Prevalence and Incidence Mapping

Malaria transmission varies sharply over short distances, so programs need local risk estimates to target bed nets, spraying, diagnosis, and treatment. Geospatial models convert incomplete point observations into continuous risk surfaces by combining survey or surveillance data with environmental, demographic, and intervention covariates. In Kenya, for example, mapped prevalence ranged from less than 1% in highland areas to more than 40% in western lowlands, illustrating why national averages are inadequate for intervention planning [7, 8].

Bayesian geostatistical models remain central to malaria mapping, while random forests and other machine learning methods can capture nonlinear relationships. Urban analyses must account for DHS coordinate displacement of 2–10 km. Spatial optimization can improve alignment with environmental covariates, although models in several African cities explained only 30%–40% of observed variation. Satellite imagery can add fine‐scale environmental information, but local validation remains necessary [7, 9].

Coupled climate–hydrology–malaria models can reproduce seasonal transmission patterns and may support early warning when linked to operational surveillance. Across these approaches, the principal contribution is not methodological complexity itself but more precise identification of where and when control resources are needed [5].

#### 3.1.2. Tuberculosis (TB) Hotspot Mapping and Spatial Autocorrelation

TB is difficult to control because underdiagnosis and incomplete notification can conceal transmission concentrated in urban settlements, mining communities, prisons, and other high‐risk locations. Spatial autocorrelation and hotspot statistics can identify clusters of notifications or diagnostic gaps, helping programs prioritize active case finding and diagnostic services. However, low notification may indicate either low incidence or poor case detection. TB maps must therefore be interpreted alongside healthcare access and surveillance completeness [10].

Geographically weighted regression can assess how associations between TB burden and poverty, education, and crowding vary by location. Overlaying TB and HIV maps can also identify areas where integrated screening and treatment are most needed. These analyses are most useful when notification, laboratory, and treatment data use consistent geographic identifiers [11, 12].

#### 3.1.3. Cholera Clustering in Humanitarian Emergencies

Cholera spreads explosively after conflict or disasters, so responders need fast, location‐specific data to stop outbreaks before they spread further. Cholera outbreaks in conflict‐affected and postdisaster settings epitomize the operational necessity of rapid geospatial surveillance in the LRS. Spatial clustering analysis using SaTScan software identified statistically significant cholera case clusters in Yemen and Somalia, enabling targeted oral rehydration therapy distribution and water quality interventions [13]. RS‐derived flood extent maps, integrated with cholera case geolocation, quantify spatial associations between inundation and disease incidence, and validate waterborne transmission pathways [5]. Predictive models incorporating rainfall anomalies (CHIRPS data), population displacement (satellite‐based enumeration), and water‐source proximity forecast cholera risks with sufficient lead time for preemptive interventions [5].

In the Democratic Republic of Congo, Google Earth imagery facilitated navigation during a polio outbreak response, demonstrating how freely available geospatial tools enhance field operations and resource allocation, even in the absence of traditional GIS infrastructure [14]. However, challenges persist: duplicate village names, reliance on unpaved roads, and scarcity of standardized addressing systems in Ghanaian settings complicate the precise geospatial analysis of typhoid fever cases, underscoring infrastructure gaps [13].

Cholera can spread rapidly after conflict, flooding, or infrastructure disruption; responders therefore need timely, location‐specific evidence. Spatial cluster detection can locate concentrations of cases, while flood extent, rainfall anomalies, displacement estimates, and water‐source proximity can identify areas requiring water, sanitation, treatment, and vaccination interventions. In humanitarian settings, these layers may support anticipatory action, although their accuracy depends on current case locations and population estimates [5, 13].

Simple tools can also provide operational value where formal GIS infrastructure is limited. During a polio response in the Democratic Republic of the Congo, Google Earth imagery supported navigation and team deployment. Conversely, duplicate place names, unpaved roads, and weak addressing systems can impede precise case mapping, as reported for typhoid surveillance in Ghana [14, 15].

#### 3.1.4. Dengue Risk Surface Generation and Environmental Correlates

Dengue risk depends heavily on local environmental conditions like rainfall and urban density, making it a strong candidate for geographic risk mapping. Dengue epidemiology in Asia and Africa exhibits a strong spatial structure driven by environmental and socioeconomic determinants that are suitable for geospatial modeling. GIS‐based dengue risk mapping in Lahore, Pakistan, integrating the Normalized Difference Vegetation Index (NDVI), temperature, and rainfall data, revealed that dengue incidence correlates with heavy rainfall (*R* ^2^ = 0.66–0.80), elevated temperature, and low vegetation cover [15]. Spatial autocorrelation analysis (Moran’s I) in Karnataka, India, identified significant clustering of dengue and chikungunya cases, with moderate positive spatial autocorrelation for dengue in specific years, indicating localized transmission dynamics [16].

Machine learning algorithms, such as random forest classifiers, support vector machines, and neural networks, predict dengue incidence from meteorological and sociodemographic variables, achieving high accuracy in urban settings, though requiring methodological adaptation for LRS data sparsity [17]. Geostatistical mapping of dengue in Bangladesh using negative binomial generalized linear mixed‐effect models accounts for zero‐inflation (underreporting) and spatial and temporal random effects through integrated nested Laplace approximation (INLA), revealing seasonal spread patterns crucial for targeting vector control [18]. Multivariate spatiotemporal approaches combining patient incidence locations, positive breeding containers, land use, and elevation in Sri Lanka have developed GIS‐based risk models that identify high‐risk hotspots, facilitating efficient resource allocation [19].

Geographic profiling techniques, originally developed for criminology, adapt to dengue control by identifying probable vector breeding sites from patient case distributions, thus enabling targeted source reduction in resource‐constrained settings [19]. Spatiotemporal diffusion pattern analysis in Thailand generates daily case maps that reveal spatial propagation, thereby informing reactive containment strategies [20].

A nationwide spatiotemporal ecological analysis of dengue in Bangladesh (2021–2025) identified urban hyperendemicity and emerging coastal per‐capita risk using government surveillance data, illustrating how routinely collected national data can reveal shifting dengue risk geographies beyond traditionally studied urban centers [21].

#### 3.1.5. Vaccine‐Preventable Diseases: Polio and Measles

Vaccination programs need to know precisely which communities are under‐immunized, since missed pockets of unvaccinated children can sustain outbreaks. Geospatial methods contributed critically to polio eradication efforts in Africa, with the WHO Regional Office for Africa (AFRO) GIS Center deploying mobile device‐based and cloud‐based tools for Auto‐Visual Acute Flaccid Paralysis (AFP) Detection and Reporting, electronic integrated supportive supervision for real‐time case geolocation, vaccination campaign monitoring, and resource dispatch [22]. Google Earth imagery enabled navigation along the Congo River during outbreak responses, enhanced vaccination team deployment, and created advocacy maps illustrating the spatiotemporal relationships between polio cases and response activities [14]. Despite the eradication of wild‐type poliovirus in 2020, the emergence of circulating vaccine‐derived polioviruses (cVDPV) and WPV1 importation from Central Asia necessitated sustained geospatial surveillance [22].

Measles spatial clustering analysis, although less extensively documented than malaria or polio, identifies vaccination coverage gaps through hotspot analysis, guiding supplementary immunization activities (SIAs) in underserved areas. Geographically weighted regression models elucidated spatially varying determinants of vaccination uptake, revealing localized barriers to healthcare access, cultural beliefs, and caregiver education amenable to community‐specific interventions [11].

### 3.2. Climate and Environmental Health Applications

#### 3.2.1. Flood‐Related Waterborne Disease Modeling

Floods create sudden spikes in waterborne disease, so mapping flood‐prone areas alongside health facility access helps target emergency response. Flooding events amplify waterborne disease transmission through the contamination of drinking water sources, displacement of populations to overcrowded temporary shelters, and disruption of sanitation infrastructure. Geospatial modeling integrating satellite‐derived flood extent (Sentinel‐1 synthetic aperture radar, MODIS surface water), precipitation data (CHIRPS), and disease surveillance records quantifies spatial associations between inundation and cholera or diarrheal disease incidence [5]. Process‐based hydrology models (WRF‐Hydro) simulate surface water accumulation and connectivity, identifying communities at elevated risk of waterborne disease outbreaks with sufficient temporal resolution for preemptive interventions [5].

In South Asia, geospatial analysis of flood‐prone areas combined with population density (WorldPop) and healthcare facility accessibility (travel‐time analysis) generates composite vulnerability indices that guide the prepositioning of medical supplies and water purification materials [23]. RS‐derived water body mapping tracks temporal changes in vector breeding habitats, informing integrated vector management strategies for flood‐associated diseases, including malaria, dengue, and Rift Valley fever [24].

#### 3.2.2. Drought‐Driven Malnutrition Hotspots

Drought‐driven malnutrition tends to concentrate in specific pastoral and agricultural regions, making geographic targeting essential for food aid. Drought‐induced food insecurity precipitates acute malnutrition crises in pastoral and agropastoral communities across the Sahel, the Horn of Africa, and Southern Africa, necessitating spatial early warning systems. Geospatial modeling combining vegetation anomalies (NDVI time series from MODIS), rainfall deficits (CHIRPS standardized precipitation index), livestock mobility patterns (mobile phone data), and anthropometric survey data (mid‐upper arm circumference screening) generates malnutrition risk maps at district to subdistrict scales [25]. These models enable the targeting of emergency nutrition interventions, community‐based management of acute malnutrition, and supplementary feeding programs to the populations most vulnerable before famine mortality escalates [25].

Spatial regression models elucidate the determinants of childhood stunting, a chronic malnutrition indicator, revealing significant associations with water access, sanitation, maternal education, and agricultural productivity [26]. Geospatially resolved estimates of child growth failure (stunting, wasting, and underweight) derived from DHS data using Bayesian hierarchical models identify persistent hotspots in rural areas of East Africa, where interventions require sustained investment [25].

#### 3.2.3. Heatwave and Mortality Risk Mapping

Heat‐related deaths are not evenly distributed, and mapping who is most exposed helps direct cooling centers and health warnings. Climate change intensifies heatwave frequency, duration, and intensity, elevating mortality risk, particularly among the elderly, outdoor workers, and populations lacking cooling infrastructure, disproportionately affecting LRS. The geospatial mapping of heat vulnerability integrates meteorological data (ERA5 temperature grids), population demographics (age structure from WorldPop), land surface temperature (LST) (MODIS, Landsat thermal bands), and urban heat island mapping (Sentinel‐2‐derived land cover) to identify high‐risk zones [27]. Spatially resolved heat‐health vulnerability indices guide targeted interventions, such as public cooling centers, behavior change communication campaigns, and occupational health regulations for outdoor labor [28].

GWR models revealed spatially varying associations between water vapor pressure and cardiovascular disease risk in Ethiopia, with significant hotspots in urban centers and arid regions, informing climate‐sensitive health planning [27]. Integration of heatwave forecasts from climate models (ERA5 ensemble predictions) with geospatial vulnerability assessments can enable the issuance of early warnings, allowing the preemptive activation of heat‐health action plans in resource‐constrained municipal contexts [27].

#### 3.2.4. RS for Environmental Health Exposure Assessment

Many LRS lack ground air‐quality monitors, so satellite data offer the only practical way to estimate pollution exposure across large areas. Satellite RS provides a scalable and cost‐effective environmental exposure assessment in LRS lacking ground‐based monitoring networks. MODIS‐derived vegetation indices (NDVI, Enhanced Vegetation Index) served as proxies for vector habitat suitability, correlating with malaria and dengue transmission intensity [24]. LST from thermal infrared bands quantifies urban heat island effects and identifies the microclimates that influence vector biology [15]. Precipitation estimates (CHIRPS, TRMM, and GPM) characterize rainfall patterns that modulate vector breeding, waterborne disease risk, and agricultural productivity [5].

Air‐quality monitoring via satellite‐derived PM2.5 estimates illuminates the spatial patterns of ambient air pollution in African and Asian cities lacking surface monitoring stations. Studies linking PM2.5 exposure data (SEDAC Global Annual PM2.5 Grids) with child mortality data (IPUMS‐DHS) found that a one standard deviation increase in outdoor PM2.5 raises under‐five mortality risk by 14% (OR: 1.14, 95% CI: 1.10–1.18). Household air pollution from solid fuel use further amplifies this risk, raising it by 37%–40% (OR: 1.37–1.40) [29]. Low‐cost air‐quality sensor networks calibrated against reference instruments expand monitoring coverage in sub‐Saharan Africa, although challenges with sensor accuracy during dry seasons (higher mean absolute error and root mean squared error) necessitate robust calibration protocols [15].

### 3.3. MCH: Mapping Inequalities in Service Coverage

#### 3.3.1. Spatial Inequality in Antenatal Care, Skilled Birth Attendance, and Postnatal Care

Access to MCH services varies widely within countries, and mapping this variation reveals which districts are being left behind. MCH indicators exhibit profound subnational inequalities across SSA, with service coverage varying by wealth quintile, education, urban–rural residence, and geographic region. Geospatial analysis of DHS cluster‐level data using spatial autocorrelation, hotspot analysis, and geographically weighted regression quantifies these disparities with policy‐relevant spatial granularity [30].

In 16 SSA countries, concentration index analysis revealed significant wealth‐based inequality in the maternal continuum of care (ANC, facility delivery, postnatal care), with pro‐rich inequalities concentrated in urban areas and among educated women [30]. Decomposition analysis attributes 40%–60% of the observed inequality to maternal education and household wealth, underscoring socioeconomic determinants amenable to targeted social protection interventions [30]. Spatial analysis in Pakistan using GWR identifies districts with extreme maternal and child mortality clustering (District Mortality Index inequality ratio of 16:1 between upper and lower deciles), with high–high clusters (Local Indicators of Spatial Association) concentrated in Baluchistan province, where healthcare access remains severely limited [31].

Capital cities have historically enjoyed primacy in MCH outcomes, but recent trends reveal erosion of this urban advantage as coverage inequalities narrow, driven by faster progress in rural areas [32]. In Eastern Africa, under‐five mortality gaps between capital cities and rural areas closed almost completely by 2015, while urban slums increasingly exhibit mortality rates comparable to or exceeding rural levels, reflecting heterogeneity within urban settings masked by aggregated urban–rural dichotomies [33]. Geospatial mapping disaggregating urban areas by wealth and neighborhood characteristics exposes hidden deprivation in periurban informal settlements, where service coverage lags [32].

#### 3.3.2. DHS Cluster‐Level Mapping for MCH

Household survey data can be mapped to reveal exactly where antenatal and delivery care coverage gaps are worst. The DHS program, covering 90 countries with standardized health, population, and nutrition indicators since 1984, provides foundational data for geospatial MCH analysis in the LRS. Cluster‐level geocoordinates, provided with intentional spatial displacement (2‐km urban, 5‐km rural, up to 10 km for 1% of rural clusters), enable the linking of health outcomes with geospatial covariates, such as environmental factors, healthcare accessibility, and socioeconomic indices, while protecting respondent confidentiality [11].

Bayesian spatial regression models estimate ANC, skilled birth attendance, and postnatal care coverage at fine spatial scales, revealing hotspots of low utilization that require intensive outreach [11]. Multivariate geospatial analysis of unskilled birth attendance in 30 SSA countries identified random spatial variations with main hotspots in Chad and cold spots in South Africa and the DRC, associated with rural residence, low wealth, limited education, maternal age, and distance to health facilities [11]. Spatial Poisson point process models incorporating patient incidence locations, facility positions, road networks, and population distribution optimize resource allocation to improve maternal healthcare access [23].

GWR models examining nonadherence to WHO’s 8‐contact antenatal care model in Ghana revealed that districts in northeastern and southwestern regions, particularly those with low community socioeconomic status and lack of health insurance, exhibit elevated noncompliance rates (17%–34% higher risk), requiring targeted policy interventions [34].

#### 3.3.3. Accessibility Modeling: Travel Time to Health Facilities

How long it takes a mother to reach a clinic strongly affects whether she gives birth safely, making travel‐time mapping a practical planning tool. Geographic accessibility to health facilities quantified as travel time to the nearest facility providing obstetric, pediatric, or immunization services a critical determinant of MCH service utilization in LRS. Geospatial accessibility modeling integrates road networks (OpenStreetMap), elevation (SRTM digital elevation models), population distribution (WorldPop), and geocoded health facility databases to compute travel‐time surfaces using least‐cost path algorithms [23].

In Mozambique, the nearest‐neighbor method analysis of spatial access to continuous maternal and perinatal healthcare services (CMPHS, encompassing ANC, institutional delivery, and postnatal care) revealed that while 80.5% of the population resides within catchment areas of facilities offering individual service packages, only 21% in 2.69% of land areas (including Maputo) can access timely, integrated CMPHS, exposing profound equity challenges [23]. Overlap analysis identifies priority districts where upgrading existing facilities to provide comprehensive care yields the greatest population impact compared to constructing new facilities.

The spatial optimization of facility placement using location‐allocation algorithms minimizes population‐weighted travel time, thereby informing evidence‐based health facility expansion plans [35]. Dynamic accessibility models incorporating transportation mode (walking, bicycle, motorized), seasonal road passability during rainy seasons, and opportunity costs of travel time provide a nuanced understanding of access barriers disproportionately affecting poor rural women [23].

### 3.4. NCDs: Mapping Emerging Epidemics

#### 3.4.1. Spatial Patterns of Hypertension and Diabetes

Chronic diseases like hypertension are rising fast in cities, and mapping where cases cluster helps target screening programs. NCDs, particularly cardiovascular diseases and diabetes, increasingly burden the LRS health systems as epidemiological transitions progress. Geospatial mapping of NCD prevalence, risk factors, and unmet health needs provides spatially targeted opportunities for prevention and care interventions [12].

Population‐based disease surveys in KwaZulu‐Natal, South Africa, coupled with novel health needs scoring (categorizing individuals as diagnosed and controlled, diagnosed but suboptimally controlled, diagnosed but not engaged in care, or undiagnosed) and spatial clustering analysis, revealed distinct geographic patterns for HIV, diabetes, and hypertension. HIV unmet needs cluster in southern urban and peri‐urban areas, whereas diabetes and hypertension unmet needs (diagnosed but uncontrolled) are concentrated in rural central and northern regions, requiring differentiated service delivery models [12]. Multivariate clustering identified significant spatial overlap of all three diseases among individuals with undiagnosed and uncontrolled conditions, highlighting opportunities for integrated screening and chronic disease management programs [12].

Geographically weighted regression models examining cardiovascular risk in Ethiopia identified statistically significant hotspots in Addis Ababa, Benishangul Gumuz, and multiple other regions, with water vapor pressure emerging as an explanatory climatic variable for geographic variations in 10‐year CVD risk [27]. This spatial heterogeneity underscores the need for context‐specific risk reduction strategies to address locally salient determinants [27].

#### 3.4.2. Environmental Exposure Modeling: Air Pollution and Health

Air pollution is a growing but poorly measured health threat in many cities, and geographic mapping helps identify the worst‐affected neighborhoods. Air pollution, particularly fine particulate matter (PM2.5), has emerged as a leading environmental health risk in LRS, driven by biomass burning, vehicular emissions, industrial activities, and desert dust. Geospatial analysis linking satellite‐derived estimates of PM2.5, with health outcomes, quantifies disease burdens and identifies priority areas for air‐quality interventions [29].

A cross‐country study across 41 LRS in South Asia and SSA, integrating SEDAC Global Annual PM2.5 Grids with IPUMS‐DHS mortality data (1998–2019), demonstrates that one standard deviation increase in outdoor PM2.5 elevates the under‐five mortality risk by 14%, while household air pollution from solid fuel use increases the risk by 37%–40%, underscoring the dual burden of ambient and indoor air pollution [34]. Multilevel logistic regression models revealed spatially heterogeneous associations, with the impacts of PM2.5, most pronounced in regions with high baseline pollution (South Asia and West Africa) [29].

In West Africa, spatiotemporal analysis revealed daily average PM2.5 concentrations of 40.9 μg/m^3^ in cities such as Lagos, Abuja, and Bamako eight times the WHO guidelines with pollution driven by desert dust, biomass burning, and urbanization. Low‐cost sensor networks deployed in sub‐Saharan Africa demonstrate the feasibility of expanding the monitoring coverage, although challenges with sensor accuracy during dry seasons (higher mean absolute error and root mean squared error) necessitate robust calibration protocols [36].

#### 3.4.3. Road Traffic Accident Mortality

Traffic deaths cluster on specific roads and districts, and mapping these patterns helps target road safety investment. Road traffic accidents represent a significant and geospatially mappable noncommunicable health burden in LRS. A district‐level geospatial analysis of road traffic accident mortality in Bangladesh (2022–2024) using routinely collected government data revealed distinct spatial and spatiotemporal clustering patterns, demonstrating the value of government administrative data for identifying high‐risk districts and informing targeted road safety interventions [37].

### 3.5. Population Mobility and Migration: Geospatial Surveillance in Dynamic Contexts

#### 3.5.1. Internally Displaced Persons, Refugees, and Humanitarian Settings

Refugee and displaced populations move quickly and unpredictably, making real‐time location tracking essential for disease control. Forced displacement creates transient, spatially concentrated populations that experience elevated infectious disease risk due to overcrowding, inadequate sanitation, disrupted healthcare access, and psychosocial stressors. Geospatial methods enable enumeration, health needs assessment, and surveillance in dynamic contexts where traditional censuses and health information systems fail [6].

Public health surveillance systems in refugee settlements in Uganda (Bidibidi, Adjumani, Kiryandongo, Rhino Camp) demonstrate the feasibility of integrated disease surveillance and response (IDSR) in humanitarian settings, although the evaluation reveals challenges: only 55% case detection, parallel implementation of partner reporting systems fragmenting data, and 49% data analysis capacity [6]. Recording (79%) and reporting (81%) performance exceeded analysis and response (34%), indicating technical capacity gaps that are suitable for training interventions [6].

Satellite imagery‐based methods for the rapid estimation of displaced populations, validated across refugee camps and IDP settlements in Bangladesh, Chad, DRC, Ethiopia, Haiti, Kenya, and Mozambique, demonstrate the feasibility of structure counting and population extrapolation from very high‐resolution imagery, providing real‐time situational awareness when ground‐based enumeration is inaccessible [19]. Computer vision models applied to satellite imagery of Ukrainian cities detect car count changes, enabling the estimation of internal displacement patterns during the 2022 Russia–Ukraine conflict with accuracy validated against administrative data [38].

Spatial modeling of health service utilization in host communities proximate to refugee and IDP camps in 30 SSA countries (1986–2016) reveals counterintuitive positive effects: Women living within 25 km of displaced populations exhibit increased institutional births, with stronger effects among the poorest mothers, attributable to humanitarian infrastructure spillovers benefiting host populations [39]. This finding challenges common misconceptions regarding competition for services and underscores opportunities for integrated programming [39].

## 4. Challenges of Geospatial Epidemiology in LRS

### 4.1. Data Limitations

Geospatial epidemiology in LRS is fundamentally constrained by weaknesses in the underlying data ecosystems. Data quality issues such as incomplete reporting, geocoding errors, and unstable denominators are not unique to LRS and are documented in high‐income settings as well; however, they are more severe and more consequential in LRS, where alternative data sources, redundant systems, and rapid correction mechanisms are largely absent. Routine surveillance systems, typically built on DHIS2‐based health management information systems (HMIS), are characterized by incomplete reporting, delayed submission, and variable adherence to case definitions, particularly in peripheral facilities and humanitarian contexts [1]. Evaluations from Ethiopia and Malawi show that while 70%–80% of facilities submit reports on time, only around two‐thirds achieve acceptable agreement (±10%) between register data and DHIS2 records, with lower performance in rural health centers and private facilities [40]. In refugee settlements in Uganda, only ∼55% of notifiable disease cases were captured by the surveillance system, and less than half of the districts regularly conducted subnational data analysis [6]. These gaps introduce substantial spatial bias; under‐resourced districts and facilities with the greatest disease burdens often contribute to worst‐quality data, leading to systematic underestimation of risk in precisely the locations where spatial decision support is most needed.

Inconsistent or incomplete geocoding of health facilities and data collection sites further undermines spatial analyses. Master facility lists in several African countries exhibit substantial missingness and positional inaccuracy, particularly for remote, newly established, and informal‐sector facilities [41]. Comparative studies between master lists and GPS‐verified facility locations reported median positional errors of 150 m, with up to 10% of the facilities displaced by > 1 km [41]. In practice, these inaccuracies distort travel‐time modeling and misrepresent service catchment areas. This weakens the validity of accessibility analyses that guide facility placement, emergency referral networks, and outreach planning [23]. Efforts to establish spatially enabled health registries in Uganda demonstrate that leveraging “implicit” spatial data pools, such as linking patient IDs to facility‐level geocodes and administrative boundaries, can partially compensate for missing coordinates, but require substantial local informatics capacity and cross‐sectoral collaboration [42].

The intentional spatial displacement of DHS and MICS cluster coordinates represents a distinctive methodological challenge. While necessary for confidentiality, a displacement of 2 km in urban and 5–10 km in rural clusters disrupts the alignment between health outcomes and fine‐scale environmental covariates, such as land cover, vector habitats, and localized pollution sources [11]. Malaria risk mapping studies in Dakar, Dar es Salaam, Kampala, and Ouagadougou applied spatial optimization algorithms to “relocate” clusters within plausible buffers. Even with this correction, the resulting models explained only about 30%–40% of the observed variation in malaria prevalence, indicating residual bias from displacement and cluster‐level aggregation [7]. Similar issues arise in geographically weighted regression analyses of antenatal care and skilled birth attendance, where displacement reduces the ability to detect neighborhood‐level effects, particularly in dense urban settings with strong intraurban heterogeneity [34]. Failure to acknowledge these uncertainties risks overinterpretation of apparent hotspots or “coldspots” based on data with inherent positional noise.

Open‐data policies offer a partial remedy to these data constraints by enabling independent verification, secondary analysis, and broader research participation beyond the original data‐generating institution. Public release of routinely collected government health and administrative data allows researchers, civil society, and international partners to validate spatial patterns, triangulate findings across sources, and identify gaps that a single agency might overlook. Bangladesh illustrates both the promise and the limits of this approach: Government‐held administrative datasets, including road traffic accident records and disease surveillance data, have been made accessible enough to support district‐level geospatial analyses of mortality and transmission patterns, demonstrating the research value of routinely collected public data. However, access remains inconsistent across sectors, data granularity is often restricted to administrative units rather than finer spatial resolutions, and formal open‐data governance frameworks are still developing. Other LRS could draw on this example by establishing standardized, tiered data‐sharing protocols—publicly releasing aggregated indicators while restricting individual‐level records—thereby balancing transparency with privacy protection.

A further, often‐underappreciated limitation is the absence of reliable population denominators at appropriate spatial and temporal scales. Many LRS lack recent censuses, and gridded population estimates (e.g., WorldPop) rely on modeled disaggregation of outdated census data using satellite imagery and ancillary covariates [43]. Validation studies underscore that errors are greatest in informal settlements, periurban fringes, and areas affected by displacement, where disease risk and service gaps are the largest [43]. In humanitarian emergencies, denominators are highly fluid; camp populations can change by tens of thousands within weeks, and standard census‐based denominators rapidly become obsolete [6]. Satellite‐based structure counts and car detections can approximate the population size, but these approaches require careful calibration and assumptions regarding occupancy rates [19]. Surveillance metrics that depend on accurate denominators, incidence rates, coverage estimates, case fatality ratios, and uncertainty in population counts propagate directly into geospatial models, complicating prioritization and evaluation.

Without strategic investments in surveillance quality, geocoding completeness, and population enumeration, geospatial analyses risk reinforcing existing inequities by providing the most precise insights where data are already strong and the weakest insights where needs are greatest.

### 4.2. Technical and Infrastructure Barriers

Technical and infrastructural constraints present a second major barrier to the routine use of geospatial epidemiology in LRS. Limited GIS and spatial statistics capacity within ministries of health and national public health institutes means that sophisticated analyses are typically outsourced to academic partners or international agencies, creating dependency and hindering institutionalization [4]. Case studies from Uganda and other African countries reveal that while geospatial tools are used during vertical campaigns, such as polio SIAs supported by the WHO AFRO’s GIS center, few national programs maintain permanent GIS units with dedicated staff, career pathways, and budget lines [22]. This leads to fragmented project‐based applications rather than the sustained integration of spatial intelligence into routine surveillance and planning.

Outdated information technology infrastructure compounds these human resource gaps. Many district health offices operate with intermittent electricity, limited server capacity, and unreliable Internet connectivity, constraining the use of web‐based platforms, cloud computing, and real‐time dashboards [44]. Large raster datasets (e.g., Sentinel‐2 imagery, CHIRPS rainfall) and computationally intensive spatial models (e.g., Bayesian disease mapping using INLA) often exceed the processing capacity of standard office hardware. Analysts are therefore forced to either scale back their analytical ambitions or rely on external cloud platforms, which may conflict with national data sovereignty requirements. In practice, this favors relatively simple methods, such as choropleth mapping and basic hotspot analysis, over advanced approaches such as spatially explicit machine learning, integrated climate–health modeling, and high‐resolution accessibility modeling.

Emerging AI and machine learning applications in geospatial health further expose the infrastructure disparities. Deep learning architectures for satellite imagery classification or high‐resolution PM2.5 prediction typically assume access to GPUs, stable high‐bandwidth connections, and robust data storage—resources that are often unavailable in LRS institutions. Editorial analyses of AI for sustainable development emphasize that without targeted capacity building and infrastructure investment, the benefits of geospatial AI will remain concentrated in high‐income research institutions, exacerbating the “AI divide” in global health [45].

Technical barriers do not merely slow adoption; they actively shape the methodological repertoire in LRS, biasing practice toward low‐complexity tools, and limiting the realization of the full potential of geospatial epidemiology for precision public health.

### 4.3. Ethical Considerations

The increasing granularity and interoperability of geospatial health data raise complex ethical questions in LRS, particularly regarding privacy, confidentiality, and group‐level harm. Concerns about reidentification, geoprivacy, and data governance are similarly debated in high‐income countries with strong data protection regimes; what distinguishes LRS is the frequent absence of comprehensive legal safeguards, oversight bodies, and enforcement mechanisms to manage these risks once they materialize. While many LMICs lack comprehensive data protection legislation, vulnerable populations, including people living with HIV, key populations, refugees, and undocumented migrants, face heightened risks from unauthorized disclosure or misuse of location‐linked health information [46]. Frameworks for digital health data governance in LMICs highlight that even deidentified datasets can present reidentification risks when combined with detailed geolocation, demographic, or temporal information, especially in sparsely populated or stigmatized communities [47].

The geoprivacy literature underscores that spatial masking techniques, such as jittering coordinates, aggregation to larger areal units, or generating synthetic populations, can mitigate reidentification risk but may not fully prevent harm arising from ecological stigmatization [48]. Mapping HIV or TB hotspots at the neighborhood or village level may lead to discrimination, reduced property values, or targeted policing, even if no individual can be directly identified [12]. Conversely, excessive anonymization can undermine the actionability of maps for local decision makers. Balancing these tensions requires context‐sensitive disclosure policies, explicit community engagement, and transparency regarding data transformations to protect privacy [46].

Issues of data ownership, sovereignty, and equity further complicate geospatial research on the LRS. Empirical reviews reveal persistent under‐representation of LMIC‐affiliated lead or senior authors in high‐impact medical journals in studies largely based on LMIC data [49]. Data‐sharing initiatives, while essential for scientific progress, risk reproducing extractive patterns where data collected in LRS are exported to high‐income institutions for analysis with limited local benefits [50]. Open science guidelines for global health emphasize the need for governance arrangements that couple open data with capacity strengthening, local control over access decisions, and benefit‐sharing mechanisms [51]. For geospatial data, in particular, questions arise about who decides which spatial resolutions can be shared, under what conditions, and how risks to communities are weighed against global public health benefits.

Ethical governance of geospatial health data in LRS must move beyond narrow notions of individual consent and anonymization to encompass community harm, power asymmetries in data use, and principles of data justice and sovereignty.

The integration of national identification or health‐tracking systems with individual‐level geospatial data carries substantial privacy, security, and human‐rights risks, particularly in South Asia, Southeast Asia, and Africa, where identity politics, religious persecution, political instability, and varying degrees of state control are salient considerations. Such linked datasets could be accessed or exploited by conflicting parties or hostile actors through hacking or unauthorized access, particularly in countries experiencing political or military conflict. The potential operational benefits of ID‐linked geospatial health data must therefore be weighed against these risks, with strong governance safeguards required for data linkage, storage, access control, and permitted secondary uses.

### 4.4. Institutional and Governance Challenges

Institutional fragmentation and weak governance frameworks limit the translation of geospatial innovations into sustainable health systems. Data‐sharing policies across ministries (health, interior, planning, and environment) are often outdated, inconsistent, or absent, resulting in siloed geospatial datasets held by cadastral agencies, national statistics offices, meteorological departments, and disease control programs with limited interoperability [4]. Consequently, analysts seeking to integrate population, facility, environmental, and climate layers for comprehensive risk mapping frequently navigate protracted, ad hoc negotiation processes rather than streamlined, policy‐based access channels.

Donor fragmentation exacerbates this landscape. Vertical global health initiatives (e.g., HIV, TB, malaria, polio, and nutrition) frequently establish their own data collection platforms, GIS tools, and dashboards, which may not integrate with national DHIS2 instances or national spatial data infrastructure (NSDI) efforts [4]. Evaluations of routine data systems in Africa consistently report parallel reporting channels for donor‐funded programs, contributing to the duplication of effort, staff fatigue, and inconsistent denominators across platforms [44]. In refugee‐hosting countries, separate health information systems for refugees (often supported by UN agencies) and host populations further fragment surveillance, undermining the comprehensive geospatial analyses of service access and disease burden [52].

Finally, relatively few LRS have explicit national geospatial strategies that position health as a core sector within the NSDI. Where NSDIs exist, they may focus on land administration, transport, and environmental management, with limited engagement from health ministries and little attention paid to privacy and ethics in health geodata [53]. This absence of strategic alignment means that investments in base mapping, addressing systems, and cadastral modernization are rarely optimized for health applications, despite their potential to transform geocoding, household enumeration, and service accessibility modeling [53].

### 4.5. Limitations of This Review

As a narrative review, this work is subject to potential selection bias and subjectivity in the identification, inclusion, and interpretation of the literature. No formal quality appraisal or risk‐of‐bias scoring was applied, and the search was not exhaustive in the manner of a systematic review. This review therefore represents a thematic and interpretive synthesis of the literature rather than a systematic evidence‐mapping exercise.

## 5. Policy Implications

The challenges described in Section 4 point to actions for three groups: ministries of health, donors and technical partners, and the national institutions and researchers that govern and use geospatial health data. Each recommendation below responds directly to a constraint identified in the preceding sections.

For ministries of health, the priority is to close the data and capacity gaps described in Sections 4.1 and 4.2. Ministries should verify and update facility coordinates within national master facility lists, because positional errors distort travel‐time and catchment analyses. They should establish permanent geospatial teams with recurrent budgets and career pathways, rather than relying on campaign‐based or externally led analyses. They should also embed practical use cases within program guidelines, including vaccination microplanning, outbreak investigation, and facility‐access analysis, so that spatial evidence informs routine decisions rather than isolated projects.

For donors and technical partners, the priority is to reduce the fragmentation described in Section 4.4. Donors should fund interoperable, country‐owned systems that integrate with national DHIS2 instances, rather than parallel disease‐specific platforms. Support for imagery, cloud services, and advanced models should include local hardware, connectivity, protected staff time, and national analytical leadership, reflecting the infrastructure constraints outlined in Section 4.2. Data‐sharing requirements should combine FAIR principles with geoprivacy, community engagement, and national data sovereignty.

For national governments and researchers, the priority is to address the ethical and governance risks described in Sections 4.3 and 4.4. Governments should position health as a core sector within the NSDI and adopt tiered data‐sharing protocols that publicly release aggregated indicators while restricting individual‐level and identity‐linked records [53]. Any linkage of geospatial health data with national identification or civil registration systems should proceed only under clear legal safeguards governing access, storage, and secondary use [46, 47]. Researchers should report positional error, coordinate displacement, and denominator uncertainty alongside spatial estimates and should support local leadership and authorship in studies that use data from LRS [49].

## 6. Conclusion

Geospatial epidemiology can reveal local disease risk, service gaps, and environmental exposure that national averages conceal. Across malaria, dengue, maternal health, chronic disease, and humanitarian surveillance, its central contribution is to direct limited resources toward specific populations and places.

Its reliability is limited by incomplete surveillance, inaccurate coordinates, displaced survey locations, uncertain denominators, and weak institutional capacity. Ethical and governance failures can also expose vulnerable communities or transfer control of LRS data to external institutions. These constraints should be treated as core design considerations rather than afterthoughts.

The immediate priority is to strengthen routine information systems, verified facility registries, population estimates, and country‐owned geospatial teams. Artificial intelligence and climate–health data integration may improve early warning, but only when models are transparent, locally validated, and linked to feasible response protocols.

Geospatial epidemiology will support health equity only when spatial evidence becomes a routine, governed, and locally sustained public health function. The goal is not more maps, but better decisions for communities that conventional surveillance often misses.

## Author Contributions

Conceptualization: Yusuf Hared Abdi.

Methodology: Yusuf Hared Abdi and Sharmake Gaiye Bashir.

Formal analysis: Yusuf Hared Abdi and Yakub Burhan Abdullahi.

Resources: Sharmake Gaiye Bashir and Naima Ibrahim Ahmed.

Data curation: Yusuf Hared Abdi and Mohamed Sharif Abdi.

Writing–original draft: Yusuf Hared Abdi.

Writing–review and editing: Sharmake Gaiye Bashir, Yakub Burhan Abdullahi, Mohamed Sharif Abdi, and Naima Ibrahim Ahmed.

Project administration: Yusuf Hared Abdi.

Validation: Sharmake Gaiye Bashir and Naima Ibrahim Ahmed.

## Funding

This study did not receive any specific funding.

## Disclosure

All authors have read and approved the final version of the manuscript. Yusuf Hared Abdi (Corresponding Author) had full access to all of the data in this study and takes complete responsibility for the integrity of the data and the accuracy of the data analysis.

## Ethics Statement

Ethical approval was not required for this study because it is a narrative literature review that analyzes previously published, publicly available, deidentified data and findings. No primary data collection involving human participants was conducted, and no individual patient data are reported.

## Consent

The authors have nothing to report.

## Conflicts of Interest

The authors declare no conflicts of interest.

## Data Availability

The authors confirm that the data supporting the findings of this study are available within the article.
